# Study smart – impact of a learning strategy training on students’ study behavior and academic performance

**DOI:** 10.1007/s10459-022-10149-z

**Published:** 2022-08-23

**Authors:** Felicitas Biwer, Anique de Bruin, Adam Persky

**Affiliations:** 1 Department of Educational Development and Research, School of Health Professions Education, Maastricht University, Maastricht, The Netherlands; 2 Division of Pharmacotherapy and Experimental Therapeutics, Eshelman School of Pharmacy, University of North Carolina, Chapel Hill, NC USA

**Keywords:** Learning strategies, Study skills, Desirable difficulties, Academic performance, Metacognitive knowledge

## Abstract

Recent research shows the importance to teach students the self-regulated use of effective learning strategies at university. However, the effects of such training programs on students’ metacognitive knowledge, use of learning strategies, and academic performance in the longer term are unknown. In the present study, all first-year pharmacology students from one university attended a learning strategy training program, i.e., the ‘Study Smart program’, in their first weeks. The 20% (n = 25) lowest scoring students on the first midterm received further support regarding their learning strategies. Results showed that all students gained accurate metacognitive knowledge about (in)effective learning strategies in the short- and long-term and reported to use less highlighting, less rereading, but more interleaving, elaboration, and distributed practice after the training program. Academic performance was compared to the prior cohort, which had not received the Study Smart program. While in the previous cohort, students in the top, middle, and bottom rank of midterm 1 stayed in these ranks and still differed significantly in the final exam, students in the Study Smart cohort that received the training program improved throughout the year and differences between ranks were significantly reduced. A learning strategy training program including a remediation track for lower performing students can thus support students to study more effectively and enhance equal chances for all students at university.

All health sciences students are required to learn and retain a high amount of information during their undergraduate study. Furthermore, they are required to retrieve and apply this knowledge during their studies (e.g., in exams) but also to treat patients in their later working life. Students need to plan, monitor, and control their learning in a self-regulated way, mostly outside the classroom during self-study (Broadbent, 2017). As such, self-regulated learning and using effective learning strategies for long-term learning are essential factors contributing to lifelong learning and student success (Richardson et al., 2012). Without a solid understanding and knowledgebase, students will struggle to transfer and apply their knowledge in a different context. For this reason, effective learning strategies for long-term learning are essential. However, many students do not know what are effective learning strategies (Bjork et al., 2013) and often use passive and shallow learning strategies to prepare for their next exam, such as re-reading their notes or summarizing (e.g., Persky & Hudson, 2016). These strategies, however, have been proven to be ineffective regarding long-term retention and understanding (Bjork et al., 2013; Hartwig & Dunlosky, 2012; Karpicke et al., 2009). Often, information is forgotten soon, and students need to spend time and effort to re-learn the information. Hence, there is a high need for training programs to support effective learning strategies among health sciences students to prepare them well for their future work.

Research in cognitive and educational sciences has shown the effectiveness of so-called *desirable difficulties* for long-term retention (Bjork & Bjork, 2014; Dunlosky et al., 2013). Desirably difficult learning strategies create difficulties and cost more effort during the initial learning phase, but enhance retention and understanding in the long-term. This is for example the case in retrieval practice or distributed practice (for reviews about these strategies, see Adesope et al., 2017; Cepeda et al., 2006; Delaney, Verkoeijen, & Spirgel, 2010). Research in cognitive psychology has demonstrated that practicing retrieval of information from memory (e.g. by answering test questions) produces better long-term retention compared to repeated rereading of the material (Adesope et al., 2017; Rowland, 2014). Test-enhanced and distributed learning are also highly effective in medical education contexts and effects transfer to clinical application with standardized patients (Dobson et al., 2019; Larsen et al., 2013). However, the contributions of cognitive psychological research to the practice of medical education are still limited (Schmidt & Mamede, 2020).

The reasons why most students do not use these desirably difficult learning strategies are multifaceted. First, intuitions during studying are misleading. Students tend to use immediate access to judge the effectiveness of a learning strategy. This in turn leads to an overestimation of their performance with rather passive strategies, such as rereading, and an underestimation of their performance with desirably difficult strategies, such as retrieval practice (Nunes & Karpicke, 2015). Second, students are lacking metacognitive knowledge about which learning strategies are effective and which ones are not (Hartwig & Dunlosky, 2012; Morehead et al., 2016). Myths about learning, such as the learning style myth, are misleading but still omnipresent in students and teachers (Kirschner, 2017; Kirschner & van Merriënboer, 2013; Newton, 2015). Third, the self-regulated use of effective learning strategies is challenging. Effective learning strategies for long-term learning are more effortful and benefits pay off after a delay. Putting these strategies into practice requires deliberate practice and a behavior or habit change over time (Fiorella, 2020). Finally, students receive either no or very little instruction on effective learning strategies and how to use them (Dunlosky et al., 2013). Competence-based curricula emphasize the acquisition of content and development of competences rather than teaching how to learn that content most effectively (Frank et al., 2010). Without formal training on the effective use of learning strategies, students are likely to follow their potentially misleading experiences during initial learning and continue to use ineffective learning strategies. In light of the abovementioned difficulties in applying effective learning strategies on one’s own, it seems highly important to teach students effective learning strategies and how to use them.

Existing training programs have generally aimed at improving students’ self-regulated learning, strategy use, and motivation (Dignath & Büttner, 2008; Dörrenbächer & Perels, 2016; Hattie et al., 1996; Nunez et al., 2011; Schuster et al., 2020; Weinstein, Husman, & Dierking, 2000) in order to ultimately foster academic performance. Most existing programs, however, focused on primary or secondary school students, or on learning strategies targeting particular domains, such as reading, writing or mathematics (Donker et al., 2014). A recent meta-analysis on self-regulated learning training programs for university students including 49 studies (Theobald, 2021) showed promising effects that training programs fostered academic performance, self-regulated learning strategies, and motivation of university students. Underachieving students benefited more from the training, probably as there was more room for improvement. Yet, it is unclear whether all students could benefit similarly from a learning strategy training program or would need continuous individualized support. Furthermore, most training programs did not address how to train learning strategies for transfer to self-study (i.e., the self-regulated use of learning strategies). The long-term effects of such training programs on sustained strategy use after the training are still unclear.

With the aim to increase students’ knowledge about effective learning strategies and ultimately support students in using these effective learning strategies, a few frameworks and training program approaches were developed in recent years (Biwer, de Bruin, Schreurs, & oude Egbrink, 2020; Biwer, oude Egbrink, Aalten, & de Bruin, 2020; Endres et al., 2021; McDaniel & Einstein, 2020; McDaniel et al., 2021). Although framed differently, these programs addressed at least one of the following components: *declarative knowledge* about effective learning strategies (which strategies are effective for long-term learning), *conditional knowledge* (knowledge about when and why a specific strategy is effective), *beliefs* about the effectiveness of strategies (by addressing the experienced-learning-versus-actual-learning paradox), *motivation* to use effective learning strategies (by formulating learning goals and an action plan), and supporting *practice* (by guided strategy practice or classroom demonstrations). In first researcher-led investigations, studies showed that these training programs increased students’ metacognitive knowledge about effective learning strategies and heightened students’ intention to use effective learning strategies (Biwer, oude Egbrink, et al., 2020; Endres et al., 2021). Open questions are, however, what effects these interventions have in the long-term, when implemented in a curriculum for all first-year students. Even though it is desirable to implement such a training for all first-year students, students might benefit differently from it. Moreover, it remains an open question whether a direct learning strategy training can support students in not only using more effective learning strategies but eventually improve their grades as well. As changing behavior is difficult, students may resist change as applying new strategies is perceived as time consuming, stressful, and leads to uncertainty in performance. Finally, research has shown that lower performing students are less metacognitively accurate and may require more support in their study strategies to develop these skills (Hacker et al., 2008). As such, a program may need to be longitudinal reinforcing concepts over time in context of actual course work, especially for lower achieving students.

The current study therefore has two main aims. First, we investigated the effect of the learning strategy training ‘Study Smart’ on students’ metacognitive knowledge as well as self-regulated use of effective learning strategies in the short- and long-term. The Study Smart program consisted of three training sessions for all first year students. In addition and following a competence-based education model, the 20% lowest performing students at the first exam received regular support on study strategies in a remediation pathway. Second, we investigated if a learning strategy training can improve academic performance when compared to the previous cohort, which did not receive a learning strategy training. If students start using more desirably difficult learning strategies enhancing retention and knowledge application, this should also become visible in exam grades testing for knowledge retention and application. The results of this study can help inform programs on how to help students develop better learning strategies and potentially use them to help students achieve mastery in a competence-based curriculum.

## Methods

### Participants

The Study Smart cohort (2020) consisted of all 126 professional (> 80% have a prior degree) students of the first-year class (PY1) enrolled at the UNC Eshelman School of Pharmacy. The average age was 23 years (*SD* = 2 years) and 71% were females. Of all 126 enrolled students that attended the Study Smart program, 111 students completed all pretest, posttest, and long-term measures. One student completed the long-term questionnaire in shorter than 1.5 min and was thus excluded from further analysis. The final sample consisted of 110 students. The control cohort (2019) consisted of 158 enrolled students, 77% were females. Students of both cohorts were nearly identical regarding their prior college GPA (3.6 out of 4 in both years) and PCAT (Pharmacy College Admission Test) standardized exam (89 in 2019 and 88 in 2020). All participants signed an informed consent to release their grades and use their data at the end of the semester. The study was approved by the ethical review board of the University of North Carolina at Chapel Hill (IRB Study #20–02,045).

### The Study Smart Program

The Study Smart Program entailed three sessions: awareness, practice, and reflection. Each session was 90 min, online due to the COVID-19 pandemic restrictions, and was led by the last author. Prior to each session, students completed some individual exercises and preparation for the next session. For an overview about the program elements, see Table 1. The training was based on the learning strategies and their effectiveness as discussed in the review by Dunlosky et al. (2013).

**Table 1:** Study Smart Outline

	Pre-Class	In-Class	Prior to Next	Components
Day 1: Awareness	1. Narrated slideshow on Desirable Difficulties	1. Goals of Study Smart 2. Categorize 8 learning strategies 3. Desirable Difficulties 4. Practice Test 5. Intro Photolog	Photolog	Declarative knowledge, conditional knowledge, beliefs
Day 2: Practice		1. Experiences and Implementation Intentions 2. Sharing experiences from practice exercise 3. Practicing two strategies together in class		Motivation, practice, conditional knowledge
Day 3: Reflection	1. Exercise study motivation (Academic goal orientation survey) 2. Reflection on perceived difficulties/challenges in using effective learning strategies	1. Plenary discussion about motivation 2. Intervision session ‘critical incident method’		Motivation, practice

The aim of the first session, awareness, was to challenge students’ prior beliefs about the effectiveness of commonly used learning strategies and to provide information about their empirical evidence. Prior to the session, students watched a narrated slideshow on the importance of desirable difficulties to prepare them for the importance of effort during studying. In-class, students shared their commonly used learning strategies. The facilitator structured this brainstorm, explained the learning strategies and asked students about their beliefs concerning the effectiveness of each learning strategy for long-term learning. Students categorized the strategies into highly effective, moderately effective, and non-effective strategies. Afterward, the facilitator explained the empirical evidence of each strategy regarding their effectiveness for long-term learning, how much training is required to use a strategy, and how to implement the strategies in the classroom setting (based on Dunlosky et al., 2013). The facilitator addressed the role of desirable difficulties, the importance of deliberate practice, and the importance of investing effort and time to become good at something. Then, the facilitator explained the testing effect and the difference between experienced learning and performance, illustrated by graphs from empirical studies (taken from Nunes & Karpicke, 2015; Roediger & Karpicke, 2006). Afterward, students reflected upon a memory of when they successfully developed a new skill through deliberate practice (e.g., sports, arts, music) in a reflective writing exercise. This exercise aimed to make students aware about the importance of effort and practice for competence development. The session ended with a short practice test consisting of seven open questions about the nature and effectiveness of the addressed learning strategies. This test aimed to strengthen the information taught in the awareness session. As homework to prepare for the next session, students were asked to keep a photolog of their study behavior in the coming week. Students were given the option to send photos via Instagram to the facilitator.

The aim of the second session, practice, was to let students practice effective learning strategies with their own learning materials. In the beginning of the session, students shared their study behavior during the previous week using their photologs. Students discussed reasons for (not) having experimented with the proposed learning strategies. This was followed by a discussion of common obstacles and strategies to overcome these obstacles. Next, students were divided into small groups (4–5 students per group) and applied either self-explanation, retrieval practice, or visualization on actual course material. After 15 min, they switched groups and applied another strategy on another set of course material. Afterward, the facilitator discussed spacing and interleaving with the students making a calendar on how and when they would study the course material for the final exam, which was approximately 10 days away. The session concluded with the students writing implementation intentions – how they will use these strategies for studying, what obstacles they expect to encounter and their strategies to encounter that obstacle.

The aim of the third session, reflection, was to address students’ study motivation and commitment. The session was split across two different days. In the first day, students completed two questionnaires: one about their learning strategies (based on the survey by Kornell & Bjork, 2007), and another one about their academic goal orientation (questionnaire by Elliot & McGregor, 2001). The questionnaire exercise intended to make students more aware about their learning strategies and study motivation and to stimulate students to reflect on what they would like to achieve with their studies. Students shared their main findings of the questionnaire and their thoughts on these with the class. In the second day, after their final examinations were completed, students reflected on the obstacles during the past week using the critical incident method (Vachon & LeBlanc, 2011). They then watched two videos of students explaining their obstacles with learning strategies and were asked to reflect on these videos. The session ended with students again making a new set of implementation intentions for the remaining part of the semester. Finally, the facilitator emphasized the importance of long-term learning.

### The remediation pathway

The 20% lowest performing students in the first midterm exam of the Study Smart cohort were additionally assigned to the remediation pathway. The remediation pathway involved developing new implementation intentions (Gollwitzer & Sheeran, 2006) on how they would study for the next examination and completing a calendar of when they would study for the next midterm examination. Students in the remediation pathway received weekly reminders of common learning strategies discussed in the Study Smart Program. They were also asked to make a recorded PowerPoint® that had them teach a (theoretical) family member about the course content they performed poorly on (Hoogerheide et al., 2019). The remaining students of the Study Smart cohort were sent reminders of common learning strategies discussed in the Study Smart Program and were given access to the other materials the bottom 20% students were provided but they were not required to complete them. However, only students in the remediation pathway completed the additional exercises and received feedback from the instructor; none of the other students actually completed the additional exercises or received feedback on study strategies.

### Measures

All measures were delivered online, using the questionnaire tool Qualtrics (Qualtrics, Provo, UT). As dependent variables, we measured metacognitive knowledge about learning strategies in the pretest, posttest, and long-term retention test. Use of learning strategies was measured via self-report in the pretest and posttest, and additionally measured in weekly surveys during the study smart intervention program, and at the time of examinations. Academic performance was measured via exam scores.

#### Metacognitive knowledge

Declarative metacognitive knowledge was measured by participants rating the effectiveness for long-term learning of the strategies highlighting, summarizing, rereading, visualization, elaboration, self-explanation, interleaved practice, distributed practice, and practice testing on a rating scale from 1 (not at all effective) to 5 (extremely effective). To measure conditional knowledge, we used seven scenario descriptions (Biwer, oude Egbrink, et al., 2020; McCabe, 2011; Morehead et al., 2016). Each scenario described two strategies with different levels of empirically supported effectiveness in a specific situation. Participants rated for each scenario the extent to which the two strategies do or do not benefit learning as measured by subsequent performance on a delayed test. They rated the effectiveness of each strategy on a scale from 1 (not at all beneficial to learning) to 7 (very beneficial to learning), with a value of four indicating a neutral evaluation (i.e., the strategy is neither rated as effective nor ineffective; Morehead et al., 2016). The scenarios described the strategies (the more effective strategy marked in italic) *testing* vs. rereading, *interleaving* vs. blocking, *spacing* vs. cramming, rereading vs. *self-explanation*, *self-explanation* vs. mental imagery, summarization with vs. *without textbook (from memory)*, and reading with vs. without highlighting (both rather ineffective).

#### Use of learning strategies

Students rated the extent to which they used the strategies on a 5-point Likert scale from 1 (never) to 5 (every time I studied). Students completed these surveys in the pretest and posttest, as well as in nine learning strategy surveys during the intervention and the semester. Due to very low completion rates of these nine surveys throughout the semester, we did not include these in our further analyses. There was no measurement of use at the long-term test, as students had no classes during that week.

#### Academic performance

We measured students’ academic performance of both cohorts via their exam grades of exam 1, exam 2 and the final exam 4. Exam 3 was not taken into consideration for this study as it was different in both years and therefore, not comparable between both cohorts. Exams included 70 multiple-choice questions (each with four answer options and only one correct answer); a maximum of 70 points could be reached per exam. Exam scores are displayed as percentage-points. Questions tested mainly knowledge application (70% of the questions), and knowledge retention (30% of the questions). Example questions of both categories are provided in the Appendix. Exam 1 and exam 2 assessed content from the weeks before the respective exam, the final exam tested knowledge of the whole course. Exam questions were about 85% the same in both cohorts, 15% was different. It has to be noted that in 2019, exams took place on campus while in 2020, exams were done online due to the COVID-19 pandemic. Exams were proctored synchronously, that means all students had two devices. On the first device, the student was monitored with videoconferencing software. On the second device, the student took the exam via a computer-based testing software (Hall et al., 2021). All examinations for both cohorts were conducted on ExamSoft, a software package that blocks internet access and access to other material on computers.

## Procedure

**Fig. 1:**
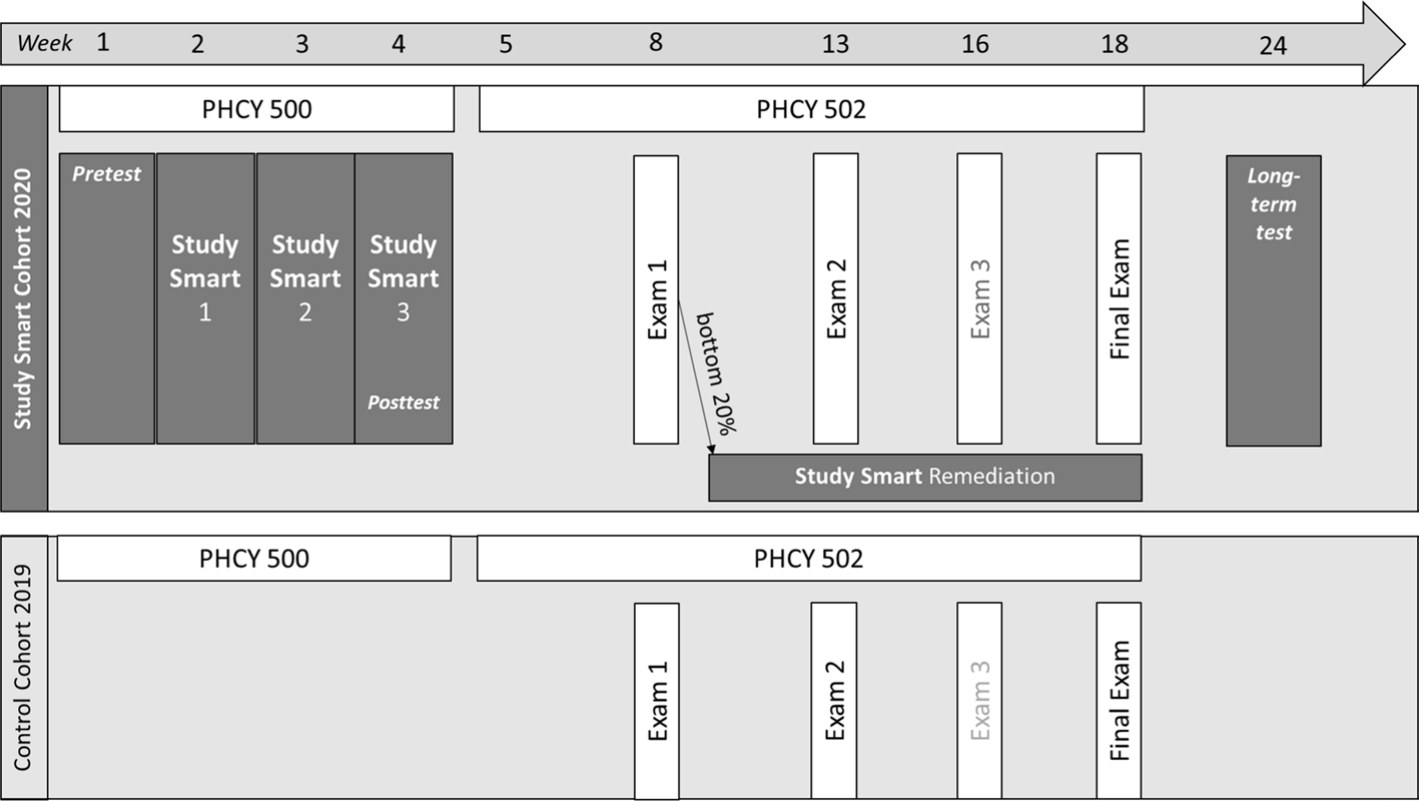
Overview of the study procedure

For an overview of the procedure see Fig. 1. This study took place during the fall semester across two core courses. The first course was a pharmacy bridging course (PHCY 500) intended to review prerequisite content essential for future learning. This course occurred during the first four weeks of the semester (i.e., August). All students of the Study Smart cohort received the Study Smart Program as mandatory part of PHCY 500. In week 1 of the course, students completed the pretest. Students attended the awareness session in week 2, the practice session in week 3, and the reflection session in week 4, and completed the posttest at the end of week 4. The second course, pathophysiology (PHCY 502), occurred in the subsequent 14 weeks (i.e., September through December). After exam 1 in PHCY 502, the 20% lowest performing students were assigned to the remediation pathway. Students who performed poorly on exams 2 and 3 were also offered this remediation pathway, but it was the cohort of exam 1 that was the primary interest for this study. In week 24, students were asked to complete the long-term retention survey. As shown in Fig. 1, the control cohort had the same courses (PHCY 500 and PHCY 502) and exams, but did not receive the Study Smart Program nor the possibility of the Study Smart remediation.

### Data analysis

An alpha level of 0.05 was used for all statistical tests. As effect size measure, we used partial eta squared with values of 0.01, 0.06, and 0.14 indicating small, medium, and large effects, respectively (Cohen, 1988). In 15% of the participants, there were missing values ranging from 1 to 13.8%, a percentage still considered acceptable (Peng et al., 2006). As indicated by Little’s MCAR test, *χ*^2^ = 1377.7, *p* = 0.214, data were missing completely at random. Therefore, listwise deletion was used to handle missing data.

To measure effects of the training on metacognitive knowledge and use of learning strategies, we conducted a repeated-measures MANOVA with time (pretest, posttest, long-term test) as repeated within-variable. Differences in academic performance between the cohorts with (2020) and without the Study Smart program (2019) were compared using a 3 (exam 1, exam 2, final exam) × 2 (cohort) × 3 (rank) three-way mixed ANOVA. Rank was used to distinguish between the lower 25 and top 25 students and the middle group (2019: n = 108; 2020: n = 76) based on the first midterm exam.

## Results

### Metacognitive knowledge

**Fig. 2:**
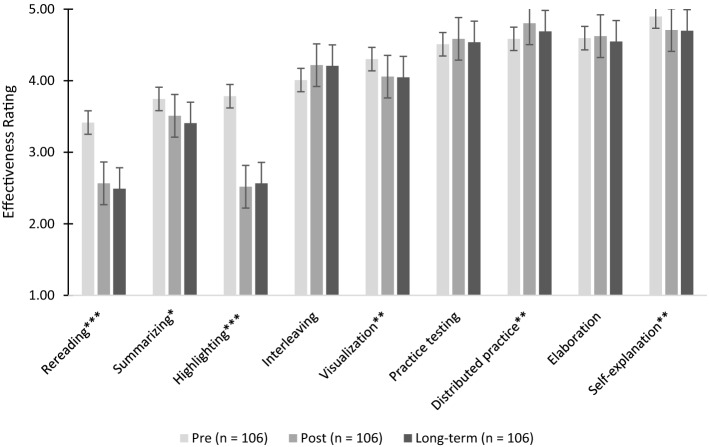
Declarative metacognitive knowledge per measurement point (pre, post, long-term). *Note*. Ratings on a scale from 1 (not at all effective) to 5 (extremely effective). Significant effects of time are marked with *p < 0.05; **p < 0.01; *** *p* < 0.001

Descriptive statistics for the effectiveness ratings at pre-, posttest, and long-term retention test are shown in the Appendix and in Fig. 2.

There was a significant main effect of time, *F *(18, 888) = 12.69; *p* < 0.001; *η*_*p*_^*2*^= 0.72. The strategies rereading, summarizing, highlighting, visualization and self-explanation were rated as significantly less effective in the post-test and long-term compared to pretest. Distributed practice was estimated as more effective in post and long-term, compared to the pretest. In the pretest, students rated all strategies as moderately or highly effective. In the posttest, the passive learning strategies highlighting, rereading, and summarizing were rated as not effective and thus more accurate with regard to scientific evidence (Dunlosky et al., 2013).

**Fig. 3:**
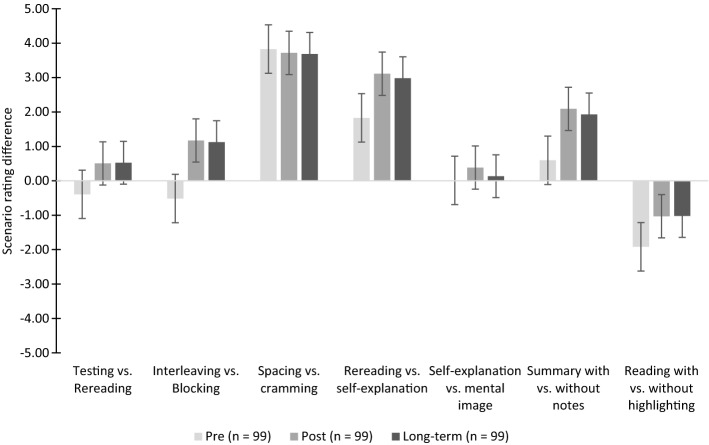
Conditional knowledge (scenario difference) per scenario and measurement point (pre, post, long-term)

Regarding conditional metacognitive knowledge, we compared the difference between effective and ineffective learning strategies across all scenarios. We expected that, at posttest and long-term, the difference between effective and ineffective strategies would be positive and higher compared to the pretest. Descriptive statistics for scenario ratings at pretest, posttest, and long-term are shown in the Appendix and in Fig. 3.

There was an overall significant multivariate effect of time, *F*(14, 85) = 9.88; *p* < 0.001; *η*_*p*_^*2*^ = 0.62. Follow-up univariate analyses showed significant time effects for the scenarios testing vs. rereading, *F*(1.8, 178.3) = 4.77, *p* = 0.012, *η*_*p*_^*2*^ = 0.05; interleaving vs. blocking, *F*(1.8, 178.9) = 16.67, *p* < 0.001, *η*_*p*_^*2*^ = 0.15; rereading vs. self-explanation, *F*(2, 196) = 22.48, *p* < 0.001, *η*_*p*_^*2*^= 0.19; summarization with vs. without textbook, *F*(2, 196) = 29.91, *p* < 0.001, *η*_*p*_^*2*^ = 0.23; and rereading with vs. without highlighting, *F*(2, 196) = 16.94, *p* < 0.001, *η*_*p*_^*2*^= 0.15. The ratings of these scenarios were more accurate in the posttest and long-term compared to the pretest.

### Learning strategy use

**Fig. 4:**
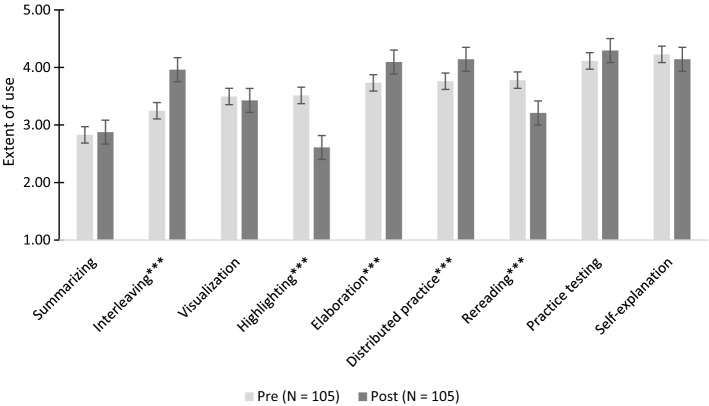
Self-reported use of learning strategies (pre, post). *Note*. Ratings on a scale from 1 (never) to 5 (every time I studied). Significant effects of time are marked with *p < 0.05; **p < 0.01; *** *p* < 0.001

The self-reported strategy use was measured pre-post. There was an overall significant multivariate effect of time, *F *(9, 96) = 11.31; *p* < 0.001; *η*_*p*_^*2*^ = 0.52, showing that strategy use differed between pre and post-test. Follow-up univariate analyses showed significant time effects for the use of highlighting, *F*(1, 104) = 52.26; *p* < 0.001; *η*_*p*_^*2*^ = 0.33, rereading, *F*(1, 104) = 24.14; *p* < 0.001; *η*_*p*_^*2*^ = 0.19, interleaving, *F*(1, 104) = 35.85; *p* < 0.001; *η*_*p*_^*2*^ = 0.26, elaboration, *F*(1, 104) = 15.85; *p* < 0.001; *η*_*p*_^*2*^ = 0.13, and distributed practice, *F*(1, 104) = 14.31; *p* < 0.001; *η*_*p*_^*2*^ = 0.12. As shown in Fig. 4 (and the Appendix), students reported to use less highlighting, less rereading, but more interleaving, elaboration, and distributed practice after the training program.

### Academic performance

**Fig. 5:**
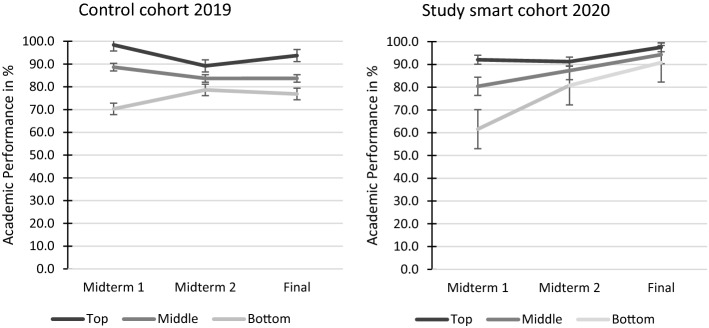
Academic Performance in % per cohort (2019 and 2020) and rank (top, middle, bottom)

For descriptive statistics see the Appendix and for a visualization of the results Fig. 5. We compared both cohorts, the control cohort 2019 and the study smart cohort 2020, using a three-way mixed ANOVA with cohort (2019 or 2020) and rank (top, middle, bottom) as between variables and exam scores over time (midterm 1, midterm 2, and final exam) as within variable.

There was a significant main effect of exam performance, *F *(2, 556) = 106.25, *p* < 0.001, *η*_*p*_^*2*^ = 0.28, showing that exam performance differed across cohorts and ranks between the different exams. There was a significant interaction effect between exam and cohort, *F *(2, 556) = 137.80, *p* < 0.001, *η*_*p*_^*2*^ = 0.31, showing that exam performance over time differed between the two cohorts. Follow-up pairwise comparisons with Bonferroni correction showed that in the control cohort 2019, exam performance only differed significantly between exam 1 and 2, with a mean difference of 1.9 points (*SE* = 0.74; *p* = 0.026). In the study smart cohort 2020, however, exam performance differed between exam 1 and 2 (*M* = 8.14, *SE* = 0.79, *p* < 0.001), exam 2 and 3 (*M* = 8.01, *SE* = 0.84, *p* < 0.001) and exam 1 and 3 (*M* = 16.15, *SE* = 0.74, *p* < 0.001) with an average increase of 12.02 points. That is, students in the study smart cohort 2020 showed more knowledge growth across exams than in the control cohort 2019.

Furthermore, the interaction between exam, cohort, and rank was significant, *F *(4, 556) = 5.67, *p* < 0.001, *η*_*p*_^*2*^= 0.039. Tests of simple effects showed that in the study smart cohort 2020, students in the top and middle group did not differ significantly in their final exam test scores, *p* = 0.078, with a mean difference of 3.2 points (*SE* = 1.45). Furthermore, the difference between the bottom and the middle group was reduced, yet still significant, *p* = 0.049, with a mean difference of 3.5 points (*SE* = 1.45). In the control cohort 2019, all ranks still differed significantly, *p*s < 0.001 in their test performance, with a mean difference of 10.0 points (*SE* = 1.4) between the top and middle group and a mean difference of 6.8 points (*SE* = 1.4) between the bottom and the middle group.

### Exploratory analyses

In exploratory analyses, we examined correlations between reported strategy knowledge and strategy use (at posttest) and academic performance at midterm 1. There was a negative correlation between the effectiveness rating of rereading and exam score on midterm 1, *r* = -0.196, *p* = 0.040 and a positive correlation between the effectiveness rating of practice testing and exam score on midterm 1, *r* = 0.218, *p* = 0.022. Regarding reported strategy use, there was a positive correlation between the reported use of practice testing and exam score on midterm 1, *r* = 0.339, *p* = 0.001.

## Discussion

This study examined whether a learning strategy training (‘Study Smart program’) implemented for all first-year students, can improve students’ metacognitive knowledge and enhance the use of effective learning strategies. Novel to the existing learning strategy intervention literature was to provide lower achieving students a remediation track to offer continuous support in the self-regulated use of effective learning strategies. Furthermore, we investigated the effect of the Study Smart program on academic performance over the course of the semester by comparing the Study Smart cohort with the previous cohort.

Regarding our first aim, we found that the Study Smart program improved declarative and conditional metacognitive knowledge not only in the short-term (Biwer, oude Egbrink, et al., 2020) but also in the long-term, up until twelve weeks after the intervention. Before the training program, students already had relatively high prior knowledge on the efficacy of practice testing, self-explanation, elaboration and distributed practice. However, students overestimated the efficacy of less effective strategies such as highlighting or rereading and gained more accurate knowledge about these strategies after the training, measured in an immediate posttest as well as long-term after twelve weeks. The conditional and declarative metacognitive knowledge changes are consistent with prior studies, although there were some nuanced differences (Biwer et al., 2020). Regarding the use of learning strategies, we found that students reported to use more effective learning strategies, such as interleaving, elaboration, and distributed practice after the training, and less non-effective strategies such as highlighting or rereading. This was, however, only measured on the post-test, due to the fact that students did not have classes in the week prior to the long-term test. In further exploratory analyses, we found that students, who, after attending the Study Smart program, reported to use more practice testing scored higher on the first midterm exam.

The second aim was to examine the effect of a learning strategy training such as the Study Smart program on academic performance. In comparison to the previous cohort, students in the Study Smart cohort improved significantly from exam to exam, with an average increase of 10 percentage points, from 79% in the first exam to 94% in the final exam. In the control cohort, students’ exam performance stayed rather stable from exam to exam, on 85%. We further examined whether and how the Study Smart program can help poorly performing students improve. From midterm 1 to midterm 2, we saw improvements in the bottom 25 students of the first midterm, who failed the first midterm with an average score around 60% to earning a B with an average score of 80% on midterm 2. This change could be attributed to differences in content (easier content on midterm 2), time effects that students became accustomed to how they were assessed and their expectations, or the reinforcement of study strategies. The first two reasons, however, are not supported by the comparison between the two cohorts. Midterm 1 and midterm 2 had the same content year to year and while students in the control cohort stayed in the bottom group, students of the bottom group in the Study Smart cohort improved significantly. In the Study Smart cohort, the bottom performing students had a positive change from midterm 1 to midterm 2 (19 percentage points) and from midterm 2 to the final exam (10 percentage points). In contrast, in the prior year, this change was much smaller (8 percentage points) from midterm 1 to midterm 2 and even negative from midterm 2 to the final exam (− 1.8 percentage points). These results are promising with regard to supporting lower achieving students in their first year to adapt to university and increase success rates. While we cannot pinpoint whether these changes are due to the fact that students used more effective learning strategies or whether the structured reflection sessions in the remediation track supported students in their planning and study motivation, it seems important to provide students more and continuous support in their self-regulated learning. This aligns with research on non-completion in higher education that showed the importance of study- or learning strategy trainings, but also of coaching and remedial training to protect for non-completion (Delnoij et al., 2020). In future research, it would be important to disentangle the working ingredients of such a remediation track to advance the support, especially needed in times of online or distance education.

The study design has several limitations. Due to the implementation in a complete cohort and ethical reasons to provide the Study Smart program to all students, a control-group design was not possible. Changes in knowledge and use of strategies pre-post might be also partly due to time effects as students develop more accurate knowledge over the year. However, as known from previous research about the experienced-learning-versus-actual-learning paradox (Kirk-Johnson et al., 2019; Nunes & Karpicke, 2015), it is quite unlikely that students gained their more accurate knowledge through experience. A related issue is that we cannot pinpoint which factors of the remediation track (e.g., individual support by the teacher, feedback on time planning, or actual increase in effective study strategies) contributed to the improvement in grades of the lower-performing students in the Study Smart cohort. Future research could examine more explicitly students’ attitudes and motivation to use more effective learning strategies over time, for example by an explanatory mixed-methods design including qualitative elements.

Second, the comparison in academic performance between the Study Smart cohort and the control cohort has to be considered in light of a different context. In 2020, the COVID-19 pandemic forced students to study completely online. All sessions were offered online, also lectures and seminars. In the year before, lectures were offered hybrid, students who lived too far away from campus already studied online, others came to campus. Nevertheless, neither the instructional approach of lectures nor the intended learning outcomes of the course changed between both cohorts. Furthermore, general performance of the Study Smart cohort was comparable to the previous cohort.

Third, the use of learning strategies was only measured via self-report pre-post, and not long-term. We cannot rule out that some students might have answered socially desirable or not completely accurate. Furthermore, students did not have an open answer option and might have used other strategies not listed in the survey. However, as shown in previous studies, an open answer option did not reveal any more new strategies not included in the list (Biwer et al., 2020). In future research on students’ learning strategy use, it would be interesting to use other ways of measuring strategy use, for example by applying experience-sampling methods or using log-data in online learning environments to gain more objective measurements of students’ learning behavior (Nett, Goetz, Hall, & Frenzel, 2012; Xie et al., 2019). Another interesting avenue for future research could be to include more long-term measures of strategy use, not only over the period of a course but over the complete first year or undergraduate program.

Fourth, this study was conducted within the context of one first-year course in pharmacy at a single university. Academic performance was measured with exam scores and exams required students to retrieve mostly factual but also conceptual knowledge and required near knowledge application. Environmental factors such as instructional approaches and the assessment culture might have influenced the way students perceived the value of the Study Smart program and the value of the proposed learning strategies. In contexts in which assessment rewards more rote memorization, strategies of cramming might be sufficient to achieve high grades and engaging in more effective, but effortful strategies might be less appealing. Many factors, such as the learning environment and students’ perceptions of assessment demands influence students’ approaches to learning and perception of the usefulness of different learning strategies (Al-Kadri et al., 2012; Palmer et al., 2019). In this study, students received direct instruction on the importance of long-term learning, and also exams asked students to retain knowledge over several weeks for the whole course. However, to what extent these results generalize to other contexts with different organizational and assessment structures is a question for further research.

The fact that students in the Study Smart cohort, and to the largest extent those in the remediation pathway, improved their academic performance demonstrates the importance of formal learning strategy instruction and providing additional, continuous support to lower-performing students. Due to higher submission rates, more and more students are being admitted to studies like pharmacy. However, students not prepared to study in a self-regulated way are likely to struggle and build shallow knowledge. Providing support in their first year on how to study more effectively is a promising way to ensure equal chances for all students in their first academic year.

## Appendix A: Example exam questions for knowledge retention and knowledge application

### Knowledge Retention

A ‘left shift’ in a total white blood cell count refers to

A. a decrease in WBC, also called leukopenia.

B. an increase in WBC count.

✓C. an increase in the percentage of immature white blood cells as part of the total WBC

D. a decrease in neutrophil percentage as part of the total WBC.

Atrial fibrillation increases the risk for

A. hypertension.

B. thrombotic stroke.

C. subdural hematoma.

✓D. embolic stroke.

Which of the following is required to assess the acid–base status of a patient?

A. pulse oximetry.

B. venous blood gas

✓C. arterial blood gas.

D. serum electrolytes.

### Knowledge Application

A 80 year-old woman recently died from dementia. At the time, she did not exhibit signs of motor fluctuation or abnormal movement. Which of the following would you expect to find on an autopsy to confirm the diagnosis of Alzheimer's?

A. deficiency of apolipoprotein E4.

B. formation of Lewy bodies in striatal dopaminergic neurons.

✓C. neurofibrillary tangles.

D. degeneration of substantia nigra neurons.

A patient presents with right-sided weakness and facial droop. A CT scan rules outs other etiologies and indicates he likely suffered an ischemic stroke. It would be most crucial for acute treatments to target:

A. comorbid hypertension and hyperlipidemia.

✓B. restoring circulation and reducing thrombosis.

C. decreasing pressure in the brain.

D. stopping the intracerebral bleed.

You are the CEO of a drug development company who wants to start developing drugs to treat cancer. Based on what you know about the pathophysiology of cancer, which of the following candidate drugs would be the most effective:

A. An antibody that inhibits the immune response.

B. A small molecular inhibitor of P53, a tumor suppressor protein that induces apoptosis.

✓C. A small molecule inhibitor of FGFR, an oncogene that promotes cell growth.

D. An antibody that inhibits BRCA2, a DNA mismatch repair enzyme.

## Appendix B: Means and standard deviations for declarative metacognitive knowledge, measured by effectiveness ratings, at pretest, posttest, and long-term, sorted by rated effectiveness at pretest

**Table:**

	Pretest (n = 106)		Posttest (n = 106)		Long-term (n = 106)		Time	
	M	(SD)	M	(SD)	M	(SD)	F(df)	*η*_*p*_^*2*^
Rereading***	3.42	1.09	2.57	0.99	2.49	0.92	F(2, 210) = 43.5***	0.29
Summarizing*	3.75	0.92	3.51	1.01	3.41	1.05	F(2, 210) = 4.45*	0.04
Highlighting***	3.78	1.00	2.52	1.00	2.57	1.02	F(2, 210) = 87.63***	0.46
Interleaving	4.01	0.98	4.22	0.97	4.21	0.89	F(1.88, 197) = 2.19	0.02
Visualization**	4.30	0.87	4.06	0.80	4.05	0.81	F(2, 210) = 4.86**	0.04
Practice testing	4.51	0.76	4.58	0.66	4.54	0.78	F(1.86, 172.5) = 0.46	0.01
Distributed practice**	4.58	0.63	4.80	0.40	4.69	0.56	F(1.85, 172) = 4.96**	0.05
Elaboration	4.59	0.61	4.62	0.58	4.55	0.66	F(2, 186) = 0.57	0.01
Self-explanation**	4.90	0.31	4.71	0.53	4.70	0.52	F(2, 186) = 7.44**	0.07

*Note*. Ratings on a scale from 1 (not at all effective) to 5 (extremely effective). Significant effects of time are marked with **p* < 0.05; ***p* < 0.01; *** *p* < 0.001.

## Appendix C: Means and standard deviations for conditional metacognitive knowledge, measured by scenario difference scores at pretest, posttest, and long-term

**Table:**

	Pretest (n = 94)		Posttest (n = 94)		Long-term (n = 94)		Time	
	*M*	*SD*	*M*	*SD*	*M*	*SD*	*F*(df)	*η*_*p*_^*2*^
Testing vs. Rereading	−0.39	2.87	0.51	3.08	0.53	2.98	*F*(1.82. 178.3) = 4.77*	0.05
Interleaving vs. Blocking	−0.52	3.48	1.17	2.84	1.12	2.85	*F*(1.84. 179.9) = 16.67***	0.15
Spacing vs. cramming	3.83	1.65	3.72	1.62	3.69	1.72	*F*(2. 196) = 0.31	0.00
Rereading vs. self-explanation	1.83	1.53	3.11	1.61	2.98	2.05	*F*(2. 196) = 22.48***	0.19
Self-explanation vs. mental image	0.01	1.22	0.38	1.52	0.13	1.22	*F*(2. 196) = 2.58	0.03
Summary with vs. without notes	0.60	1.63	2.09	1.55	1.93	1.86	*F*(2. 196) = 29.9***	0.23
Reading with vs. without highlighting	−1.92	1.43	-1.03	1.55	−1.02	1.41	*F*(2. 196) = 16.93***	0.15

*Note*. Ratings on a scale from 1 (not at all effective) to 5 (extremely effective). Significant effects of time are marked with **p* < 0.05; ***p* < 0.01; *** *p* < 0.001.

## Appendix D: Means and standard deviations for self-reported extent of use, measured pre and post

**Table:**

	Pretest (n = 105)		Posttest (n = 105)		Time	
	*M*	*SD*	*M*	*SD*	*F*(df)	*η*_*p*_^*2*^
Summarizing	2.83	1.26	2.88	1.19	*F*(1, 104) = 0.12	0.00
Interleaving***	3.25	1.08	3.96	1.00	*F*(1, 104) = 35.85	0.26
Visualization	3.50	1.26	3.43	1.13	*F*(1, 104) = 0.31	0.00
Highlighting***	3.51	1.15	2.61	1.18	*F*(1, 104) = 52.26	0.33
Elaboration***	3.73	0.98	4.10	0.83	*F*(1, 104) = 15.85	0.13
Distributed practice***	3.76	0.97	4.14	0.94	*F*(1, 104) = 14.31	0.12
Rereading***	3.78	1.04	3.21	1.07	*F*(1, 104) = 24.14	0.19
Practice testing	4.11	0.95	4.30	0.85	*F*(1, 104) = 3.04	0.03
Self-explanation	4.23	0.79	4.14	0.84	*F*(1, 104) = 0.82	0.01

*Note*. Ratings on a scale from 1 (never) to 5 (every time I studied). Significant effects of time are marked with **p* < 0.05; ***p* < 0.01; *** *p* < 0.001.

## Appendix F: Means and standard deviations for exam scores of both cohorts (2020 and 2019) for midterm 1, midterm 2 and final exam

**Table:**

		2020 (Study Smart cohort)				2019 (Control cohort)			
		Top (n = 25)	Middle (n = 76)	Bottom (n = 25)	*Total*	Top (n = 25)	Middle (n = 108)	Bottom (n = 25)	*Total*
Midterm 1	M	92.06	80.39	61.57	78.98	98.40	88.63	70.30	87.28
	SD	2.75	4.96	6.76	11.00	1.22	5.25	4.69	9.45
Midterm 2	M	91.22	87.30	80.78	86.79	89.19	83.68	78.64	83.75
	SD	4.36	5.80	6.59	6.60	6.20	7.51	7.89	7.92
Final	M	97.54	94.31	90.81	94.25	93.72	83.69	76.86	84.19
	SD	3.26	4.02	6.03	4.82	4.29	7.71	8.63	8.82
